# Autoantibodies as diagnostic biomarkers for lung cancer: A systematic review

**DOI:** 10.1038/s41420-019-0207-1

**Published:** 2019-08-05

**Authors:** Bin Yang, Xiaoyan Li, Tianyi Ren, Yiyu Yin

**Affiliations:** 1 China–Japan Union Hospital of Jilin University, Changchun, China; 2 National Institutes of Health (NIH)), Bethesda, USA

**Keywords:** Predictive markers, Cancer screening

## Abstract

Lung cancer (LC) accounts for the largest number of tumor-related deaths worldwide. As the overall 5-year survival rate of LC is associated with its stages at detection, development of a cost-effective and noninvasive cancer screening method is necessary. We conducted a systematic review to evaluate the diagnostic values of single and panel tumor-associated autoantibodies (TAAbs) in patients with LC. This review included 52 articles with 64 single TAAbs and 19 with 20 panels of TAAbs. Enzyme-linked immunosorbent assays (ELISA) were the most common detection method. The sensitivities of single TAAbs for all stages of LC ranged from 3.1% to 92.9% (mean: 45.2%, median: 37.1%), specificities from 60.6% to 100% (mean: 88.1%, median: 94.9%), and AUCs from 0.416 to 0.990 (mean: 0.764, median: 0.785). The single TAAb with the most significant diagnostic value was the autoantibody against human epididymis secretory protein (HE4) with the maximum sensitivity 91% for NSCLC. The sensitivities of the panel of TAAbs ranged from 30% to 94.8% (mean: 76.7%, median: 82%), specificities from 73% to 100% (mean: 86.8%, median: 89.0%), and AUCs from 0.630 to 0.982 (mean: 0.821, median: 0.820), and the most significant AUC value in a panel (M13 Phage 908, 3148, 1011, 3052, 1000) was 0.982. The single TAAb with the most significant diagnostic calue for early stage LC, was the autoantibody against Wilms tumor protein 1 (WT1) with the maximum sensitivity of 90.3% for NSCLC and its sensitivity and specificity in a panel (T7 Phage 72, 91, 96, 252, 286, 290) were both above 90.0%. Single or TAAbs panels may be useful biomarkers for detecting LC patients at all stages or an early-stage in high-risk populations or health people, but the TAAbs panels showed higher detection performance than single TAAbs. The diagnostic value of the panel of six TAAbs, which is higher than the panel of seven TAAbs, may be used as potential biomarkers for the early detection of LC and can probably be used in combination with low-dose CT in the clinic.

## Facts

- LC is one of the most common types of cancer and accounts for the majority of tumor-related deaths globally.
- Patients diagnosed with LC at an early-stage have a higher 5-year survival rate.
- Low-dose spiral computed tomography (CT) is the most widely used diagnostic method in clinical practice, but its the high false positive rates and cost may prevent it from becoming a routine screening method.
- Current research and studies aim to identify the possibility of the molecular makers in body fluids, like TAAbs, for the early detection of LC.

## Open questions

- Currently some TAAbs have been studied. How are they related to diagnosis and how can the appropriate TAAbs for detecting early-stage LC be selected?
- It is still worth investigating whether the different distributions of TAAbs in the body are long lasting and have high concentration in blood.
- TAAb detection combined with CT can probably be used in clinic for detection of LC in the future.
- TAAbs combined with other biomarkers like miRNAs will probably have improved diagnostic performance.

## Introduction

Lung cancer (LC) is one of the most common types of cancer and accounts for the largest number of tumor-related deaths globally. There are an estimated 705,000 cases and 569,000 deaths due to LC in China, and 214,000 cases and 168,000 deaths in US in 2012^1,2^. The overall 5-year survival rate of LC is associated with its stages at doagnosis, which is <20% as the majority of cases are diagnosed at late stages, In contrast, tumors diagnosed at stage IA have a 5-year survival rate of ~70%^3^. Therefore,early detection and immediate treatment can reduce the mortality of LC significantly. However, the detection and diagnosis of early stage LC is still a challenge, because of the lack of effective screening methods. It has been proven that sputum exfoliative cytologic examination cannot effectively reduce LC mortality^4^. In contrast, low-dose spiral computed tomography (CT) is highly sensitive at the early detection of small lung nodules and has led to a 20% reduction in LC mortality^5^, but its high false positive rates and cost may prevent it from becoming a routine screening method^4,6^.

Thus, it is necessary to develop more cost-effective and noninvasive cancer screening methods. Current research and studies aim to identify molecular makers, that could be detected in body fluids for the early detection of LC. Current diagnostic methods have concentrated on tumor-associated antigens (TAAs) markers, such as the carbohydrate antigen (CA) 125, CA19-9, carcino-embryonic antigen (CEA) and alpha fetal protein (AFP), which are effective at diagnosing LC at advanced stages^7^, but have a low sensitivity and specificity for early stage LC. However, detection of tumor-associated autoantibodies (TAAbs), which are produced by cancer cells against TAAs in blood, may become a potential cancer screening method^8^. TAAbs are more stable in peripheral blood than TAAs, and have better sensitivity and specificity. Clinical trials evaluating the diagnostic value of TAAbs have shown them to be potential diagnostic method as detective biomarkers for LC, and a series of candidates and multiplex TAAbs have been identified and analyzed.

Hence, we provided a systematic and comprehensive review and summary of the published articles that investigated TAAbs for LC detection. We reported on research results and indicators for assessing the diagnostic performance of TAAbs in the patients’ blood, and also put forward new research problems and new possibilities for future studies^9–12^.

## Search strategy

Our review was conducted according to a predefined protocol in accordance with the PRISMA statement^13^. A systematic literature search was performed to identify studies that assessed TAAbs in relation to LC. We searched Pubmed and ISI Web of Science for articles that were published from 1 January 1990 to 31 December 2018. The following combinations of search keywords were used to retrieve articles: ((lung OR pulmonary) AND (cancer OR carcinoma OR neoplasm OR tumor OR adenocarcinoma OR squamous carcinoma OR malignancy) AND (autoantibody OR antibody) AND (detection OR diagnosis OR biomarker OR marker) AND (serum OR blood OR plasma))in all fields. Duplicated articles were removed.

## Eligibility criteria

**Fig. 1:**
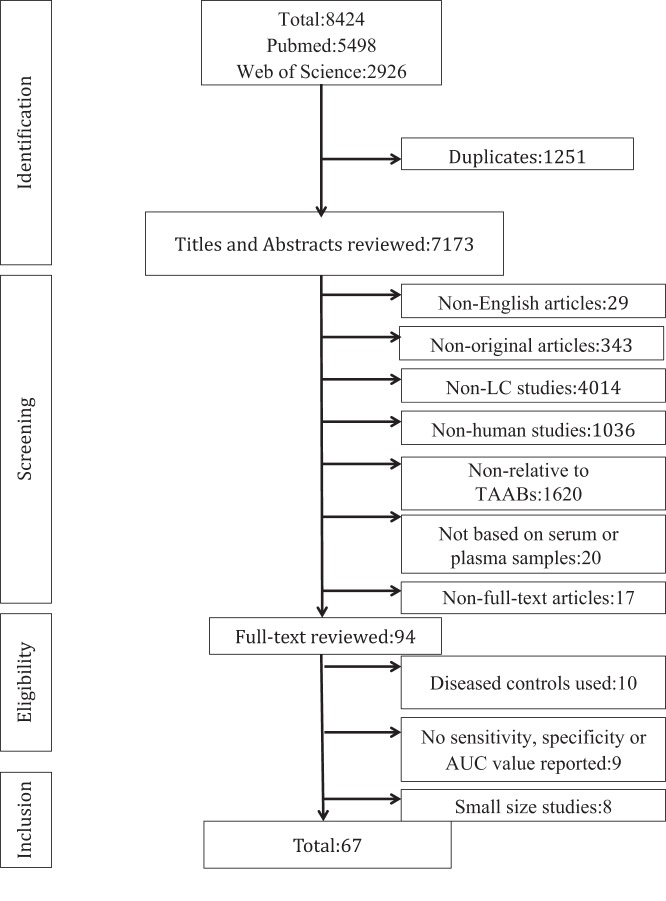
Flow process diagram showing the overview of the literature (From January 1st 1990 to December 31st 2018)

We initially read the titles and abstracts to screen the potential eligible articles, with the following exclusion criteria (Fig. 1): (1) non-English articles, (2) non-original articles (reviews, meta-analyses, or proceedings), (3) non-LC studies, (4) nonhuman studies, (5) not related to TAABs, (6) not based on serum or plasma samples, and (7) non-full-text articles. The second round of the preliminary screening involved reading the full-text of the articles, and studies with the following were excluded: (1) diseased controls used, (2) not reporting critical data or no sensitivity, specificity, or area under the curve (AUC).

## Data extraction and statistical analysis

Two reviewers (Yiyu Yin and Xiaoyan Li) independently read and extracted all the eligible articles above. Any disagreements and arguments were discussed and resolved among the authors. We extracted the first author, publication year, country, TAAs associated with the autoantibodies, study method, basic population characteristics (including size, age, sex, histological type, and tumor stage), specimen type, targeted TAAbs markers, and evaluation indicators (sensitivity, specificity, AUC, and *p*-value). Individual TAAbs with a *p*-value > 0.5 were eliminated. We use Statistical R (version 3.5.1) to calculate the mean or median ages if these statistics were not presented but the raw data were available.

## Quality assessment

The quality of each eligible article was assessed by two independent researchers according to quality assessment of diagnostic accuracy studies (QUADAS-2, www.bris.ac.uk/quadas), using Review Manager (version 5.3). QUADAS-2 contains four domains on bias and applicability of the the research question: (1) patient selection, (2) index test(s), (3) reference standard, and (4) flow and timing, and each item was assessed as “yes” or “no” or “unclear”. Applicability concerns were assessed using the first three domains as well.

## Study identification and literature search

A flow process diagram of the study search process is shown in Fig. 1. A total of 8424 potentially relevant publications were identified by the initial independent search using the search terms mentioned above, 5498 from PubMed and 2926 from Web of Science (Fig. 1). 1251 duplicate articles were removed. The titles and abstracts of 7173 articles were screened and a total of 7079 were excluded based on the exclusion criteria described above. Of the remaining 94 full-text articles, 10 were excluded because a disease control was used^14–23^, 9 were excluded because they did not have satisfied outcomes^24–32^, and 8 were excluded because of their small sample size (*n* < 10)^33–40^, Ultimately, 67 articles were included in this systematic review evaluating the diagnostic performance of TAAbs in serum or plasma for LC detection (Tables 1 and 2).

**Table 1:** Studies investigating the single autoantibody

Reference	Study	Country	Number (cases/controls)	ES (ES%)	Mean or median age(range) (controls)	Specimen	Histology	TAAbs against TAAs	Detection method	SEN% (AS)	SEN% (ES)	SPE%	AUC	*p*-value
^9^	Pei (2017)	China	50/42	29 (58.0)	66.0 ± 9.9 (45–86)	Serum	ADC(26) SCC(16) Others(8)	Cyclin B1 Survivin p53 HCC1	ELISA	20.0 32.0 18.0 22.0	NA	97.6 100 100 100	0.767 0.653 0.623 0.622	<0.001 0.012 0.042 0.045
^45^	Wang (2017)	USA	109/216	36 (33.0)	62.1 ± 10.4 (NA)	Plasma	NSCLC	ANXA1	ELISA	NA	NA	NA	NA	0.009
^79^	Dai 2017	China	242/270	NA	NA	Serum	ADC(197) SCC(45)	ENO1	ELISA	35.1	NA	80.7	0.589	0.001
^63^	Dagmar (2016)	Czech Republic	57/57	26 (45.6)	62 (30–79)	Serum	ADC(30) SCC(21) Others(6)	NY-ESO-1	ELISA	26.3	NA	96.5	NA	0.00063
^80^	Juan (2016)	China	48/27	NA	NA (35–73)	Serum	NSCLC	MUC1	ELISA	62.5	0.0	100.0	0.870	<0.001
^10^	Dai (2016)	China	90/89	30 (33.3)	67.5 ± 10.7 (41–87)	Serum	ADC(71) SCC(16) Others(3)	cyclin B1 MDM2 c-Myc p53 p16 14-3-3ζ NPM1	ELISA	13.3 14.4 15.6 16.7 21.1 22.2 37.8	NA	96.6 96.6 94.4 96.6 95.5 97.8 93.3	0.639 0.602 0.603 0.627 0.747 0.621 0.854	0.001 0.018 0.018 0.003 0.000 0.005 0.000
^46^	Natalie (2016)	USA	45/16	32 (71.1)	NA	Plasma	NSCLC	SULF2	ELISA	NA	NA	NA	NA	0.004
^64^	Yang (2015)	China	57/47	5 (8.8)	NA	Serum	SCLC	NY-ESO-1	ELISA	37.2	45.5	91.7	0.619	<0.01
^81^	Qi (2015)	China	168/97	117 (69.6)	62.5 (27–85)	Serum	ADC(123) SCC(45)	ChgA	ELISA	47.6	NA	80.0	0.688	<0.05
^82^	Pierre (2015)	France	346/41	30 (8.7)	62.08 (NA)	Serum	ADC(94) SCC(200) Others(52)	HE4	ELISA	91.0	NA	61.0	0.780	<0.0001
^54^	Manlio (2015)	Italy	201/54	68 (36.2)	NA	Serum	ADC(79) SCC(70) SCLC(13) Others(39	p53	ELISA	20.4	10.3	100.0	NA	0.005
^65^	Victoria (2015)	USA	115/115	88 (76.5)	64 (50–97)	Serum	ADC(41) SCC(45) Others(29)	NY-ESO-1	ELISA	47.0	NA	80.0	0.600	0.01007
^83^	Wang (2014)	China	272/227	121 (44.6)	57.5 ± 9.2 (NA)	Serum	NSCLC	ANXA1	ELISA	23.7	NA	90.3	0.640	<0.0001
^84^	Ma (2013)	China	264/192	74 (28.0)	58.5 (33–85)	Serum	NSCLC	CCNY	ELISA	23.5	NA	95.5	0.737	<0.001
^85^	Tetyana (2013)	USA	22/21	19 (86.4)	NA	Serum	ADC	scFvB6 scFvG1 scFvP6	ELISA	67.0 73.0 60.0	NA	80.0 67.0 73.0	0.840 0.470 0.690	0.0003 0.0136 0.0304
^86^	Dai (2013)	China	292/300	39 (13.4)	62 (40–91)	Serum	ADC(116) SCC(166) Others(10)	APE1	ELISA	38.7	39.3	NA	0.745	0.000
^47^	Ye (2013)	China	272/226	118 (43.4)	57.4 ± 9.2 (NA)	Plasma	NSCLC	CD25	ELISA	35.0	31.4	90.0	0.700	<0.001
^69^	Ying (2012)	China	190/104	21 (11.0)	61.38 (27–82)	Serum	NSCLC	IGFBP-2	ELISA	73.2	NA	60.6	0.677	<0.0001
^87^	Liu (2012)	China	275/226	NA	57.6 ± 9.2 (NA)	Serum	NSCLC	ABCC3	ELISA	18.1	NA	95.0	0.670	<0.001
^50^	Luo (2012)	China	47/43	13 (27.6)	NA	Serum	ADC(15) SCC(14) SCLC(18)	Cathepsin D	2-DE Western blot	36.2	30.8	100.0	NA	<0.05
^55^	Yongjung (2011)	Korea	82/79	NA	63.5 (55.9–70.0)	Serum	ADC(47) SCC(18) SCLC(14) Others(3)	p53	ELISA	34.1	NA	94.9	0.790	<0.001
^41^	Nada (2014)	USA	32/30	11 (34.0)	66.2 ± 0.5 (NA)	Plasma	ADC(10) SCC(11) Others(11)	M13 Phage 908 3148 1011 3052 1000	Protein Chip	84.3 84.3 90.6 90.6 90.6	NA	66.6 73.3 63.3 70.0 73.3	0.945 0.893 0.866 0.849 0.848	<0.05
^42^	Wu (2010)	China	90/90	21 (23.3)	NA	Serum	NSCLC	T7 Phage 72 91 96 252 286 290	ELISA	NA	NA	NA	0.905 0.897 0.908 0.887 0.908 0.810	<0.001 <0.001 <0.001 <0.001 <0.001 <0.001
^88^	Yao (2010)	China	93/87	38 (40.9)	60.3 (33–79)	Serum	ADC(53) SCC(23) Others(17)	DKK1	ELISA	62.0	65.8	84.0	NA	<0.05
^48^	Radostina (2010)	Bulgaria	51/52	30 (58.8)	NA	Plasma	ADC(15) SCC(36)	α-crystallin	ELISA	62.0	NA	72.0	0.712	0.001
^68^	Yusuke (2009)	Japan	91/70	45 (49.4)	70 (42–85)	Serum	ADC(54) SCC(29) Others(8)	WT1	ELISA	26.4	90.3	90.2	NA	<0.05
^49^	Huan (2018)	China	211/200	20 (9.5)	NA	Plasma	ADC(124) SCC(87)	p16a	ELISA	32.7	NA	95.0	0.818	<0.001
^89^	Maxim (2017)	Switzerland	93/94	2 (2.1)	59 (19–86)	Serum	ADC(42) SCC(22) Others(29)	BARD1	ELISA	80.0	NA	77.5	0.860	0.003
^90^	Jung (2017)	Korea	80/80	NA	68.5 ± 9.3 (NA)	Serum	NSCLC	AIMP2-DXIMP2	ELISA	NA	NA	NA	0.416 0.579	<0.05
^66^	Petra (2008)	Germany	39/40	18 (46.2)	NA	Serum	SCC(39)	27 Phage	serological spot assays	92.9	79.0	93.1	0.978	<0.05
^91^	Zhang (2017)	China	72/70	51 (70.8)	64 (37–82)	Serum	ADC(38) SCC(24) SCLC(4) Others(6)	ENO1	ELISA	80.6	NA	72.7	0.806	<0.05
^52^	Wu (2018)	China	127/127	39 (30.7)	57 (32–76)	Serum	ADC(70) SCC(57)	TOPO48	ELISA	76.0	71.8	100.0	0.990	0.001
^43^	Jie (2015)	USA	97/87	46 (47.4)	70 (62–77)	Plasma	ADC(97)	TTC14 BRAF ACTL6B MORC2 CTAG1B	ELISA	11.3 5.2 3.1 4.1 9.3	NA	97.7 97.7 97.7 97.7 97.7	NA	<0.05
^92^	Lei (2017)	China	206/99	32 (15.5)	NA	Serum	NSCLC	dickkopf-1 PepB	ELISA	58.1	76.9	85.3	0.821	0.008
^93^	Pei (2016)	China	62/43	13 (21.0)	66.0 ± 9.9(NA)	Serum	NSCLC	MDM2 c-Myc	ELISA	37.1 35.5	NA NA	97.7 97.7	0.777 0.815	0.001 0.001
^53^	Dominique (2014)	Netherlands	44/49	NA	NA (50–75)	Serum	NSCLC	Fab	SDS-PAGE, LCMS	84.0	NA	90.0	NA	<0.05
^94^	Dai (2017)	USA	90/89	30 (33.3)	67.5 ± 10.7 (41-87)	Serum	ADC(81) SCC(6) Others(3)	ECH1 HNRNPA B1	ELISA	62.2 72.2	NA NA	95.5 95.5	0.799 0.874	<0.001 <0.001
^56^	Mack (2000)	Germany	134/46	29 (21.6)	61.9 ± 10.9 (NA)	Serum	SCC(44) ADC(44) SCLC(35) Others(11)	p53	ELISA	12.6	NA	97.8	NA	<0.001
^57^	Jerzy (1998)	Poland	84/20	37 (44.0)	NA	Serum	SCC(43) ADC(27) LCC(14)	p53	IHC	22.6	40.4	NA	NA	0/002
^58^	Toshihiko (1998)	Japan	62/41	33 (53.2)	65.7 (48–85)	Serum	ADC(33) SCC(21) LCC(8)	p53	ELISA	40.3	48.5	NA	NA	0.0025
^99^	Mikio (2001)	Japan	50/130	NA	NA	Serum	ADC(32) SCC(47) SCLC(4) LCC(6) Others(27)	HSP40	ELISA	NA	NA	NA	NA	<0.001
^59^	Jassem (2001)	Poland	96/41	60 (62.5)	58 (35–86)	Serum	SCLC	p53	ELISA	27.0	25.0	97.5	NA	<0.001
^60^	Cioffi (2001)	Italy	109/80	21 (19.3)	NA	Serum	NSCLC(57) SCLC(52)	p53	ELISA	32.1	38.1	100.0	NA	NA
^61^	Monica (2002)	Italy	78/106	2 (3.6)	62.4 ± 9.3 (NA)	Serum	ADC(18) SCC(19) SCLC(3) Others(8)	p53	ELISA	12.8	0	98.1	NA	0.01
^62^	Suleeporn (2003)	Thailand	133/200	30 (22.6)	NA	Serum	ADC(59) SCC(29) LCC(4) SCLC(13)	p53	ELISA	18.8	6.7	97.5	NA	<0.001
^100^	Tsuji (1997)	Japan	67/60	NA	NA	Serum	ADC(51) SCC(9) SCLC(5) LCC(2)	TRD-L1	ELISA	55.2	NA	97.7	NA	<0.001
^102^	Dennis (2003)	USA	49/40	0 (0)	NA	Serum	ADC(14) SCC(17) LCC(1) Others(17)	HSP70	ELISA	74.7	NA	73.0	0.731	<0.001
^103^	Zhong (2004)	USA	49/40	12 (30.0)	NA	Serum	ADC(12) SCC(19) Others(18)	HSP70	ELISA	74.0	NA	73.0	0.731	0.0009
^104^	Zhong (2006)	USA	23/23	23 (100)	65.1 (51–79)	Serum	ADC(7) SCC(8) Others(8)	L1919 L1896 G2004 G1954 G1689	ELISA	82.6 87.0 82.6 82.6 82.6	82.6 87.0 82.6 82.6 82.6	78.3 87.0 65.2 87.0 65.2	0.850 0.950 0.800 0.740 0.820	<0.001 <0.001 <0.001 <0.001 <0.001
^105^	Daniel (2008)	USA	105/102	88 (83.0)	66.4 (43–85)	Serum	ADC	AZGP1	ELISA	40.0	NA	NA	NA	<0.05
^51^	Myrna (1997)	Germany	170/50	70 (41.0)	61.4 (NA)	Serum	SCLC	p53	Western blot	16.0	NA	100	NA	<0.05

*AS* all-stage, *ES* early-stage (stage I and II included), *Controls* benign diseases and normal healthy donors, *AUC* area under the curve, *SEN* sensitivity, *SPE* specificity, *ELISA* enzyme-linked immunoassay, *WB* western blotting, *ADC* adenocarcinoma, *SCC* squamous carcinoma, *SCLC* small cell lung cancer, *NSCLC* non-small cell lung cancer, *NA* not available

**Table 2:** Studies investigating the panel autoantibodies

Reference	Study	Country	Number (cases/controls)	ES (ES%)	Mean or median age(range) (controls)	Specimen	Histology	TAAbs against TAAs	Detection method	SEN%(AS)	SEN%(ES)	SPE%	AUC	*p*-value
^9^	Pei (2017)	China	60/31	NA	NA	Serum	NA	Panel 1	ELISA	65.0	NA	100	0.908	<0.001
^89^	Maxim (2017)	Switzerland	93/94	2 (2.1)	65 (28–86)	Serum	ADC(42) SCC(22) Others (29)	Panel 2	ELISA	80.0	NA	78.0	0.961	NA
^10^	Dai (2016)	China	90/89	30 (33.3)	67.5 ± 10.7 (41–87)	Serum	ADC(71) SCC(16) Others(3)	Panel 3	ELISA	68.9	73.3	79.5	0.863	<0.05
^85^	Tetyana (2013)	USA	22/21	19 (86.4)	NA	Serum	ADC	Panel 4	ELISA	80.0	NA	87.0	0.880	NA
^71^	Erin (2010)	USA	(10/10)	9 (90.0)	72 (65–86)	Serum	ADC	Panel 5	ELISA	94.8	NA	91.1	0.964	<0.05
^65^	Victoria (2015)	USA	(75/75)	23 (31.0)	68.5 (50–99)	Serum	ADC SCC Others	Panel 6	Luminex MAP	77.0	71.2	80.0	0.810	<0.0001
^72^	Boyle (2010)	UK	(145/146)	81 (55.9)	66.0 (41–87)	Serum	ADC(29) SCC(21) SCLC(22) Others(73)	Panel 7	ELISA	36.0	NA	91.0	0.710	NA
^72^	Boyle (2010)	UK	(241/88)	0 (0)	63.0 (28–87)	Serum	ADC(56) SCC(42) SCLC (70) Others(73)	Panel 8	ELISA	39.0	0.0	89.0	0.630	NA
^72^	Boyle (2010)	UK	(269/NA)	86 (32.0)	65.0 (38–87)	Serum	ADC(67) SCC(88) SCLC(73) Others(27)	Panel 8	ELISA	37.0	NA	90.0	0.640	NA
^41^	Nada (2010)	USA	(32/30)	11 (34.0)	66.2 ± 10.5 (NA)	Plasma	ADC(10) SCC(11) Others(11)	Panel 9	Protein Chip	90.0	NA	73.0	0.982	<0.05
^42^	Wu (2010)	China	(90/90)	21 (23.0)	NA	Serum	NSCLC	Panel 10	ELISA	92.2	92.2	92.2	0.956	<0.001
^43^	Wang (2015)	USA	(97/87)	46 (47.4)	70.0 (62–77)	Plasma	ADC(97)	Panel 13	ELISA	30.0	NA	88.0	NA	<0.05
^70^	Ren (2018)	China	(818/1190)	213 (26.0)	54.0 (18–91)	Serum	ADC(429) SCC(277) SCLC(91) Others(21)	Panel 11	ELISA	61.0	62.0	90.0	0.781	<0.05
^95^	Jia (2014)	China	(48/50)	NA	59.7 ± 8.7 (39–79)	Serum	NCSLC	Panel 12	Luminex MAP	NA	NA	NA	0.820	<0.05
^96^	Qiang (2018)	China	(352/129)	133 (37.8)	60.51 ± 9.41 (NA)	Serum	ADC(243) SCC(42) SCLC(47)	Panel 14	ELISA	56.5	56.4	91.6	NA	<0.001
^97^	Caroline (2010)	UK	(243/247)	90 (37%)	66 ± 9.6 (33–87)	Serum	SCLC(243)	Panel 15	ELISA	55.0	53.0	90.0	0.761	<0.001
^98^	Qiu (2008)	USA	(85/85)	NA	NA	Serum	NSCLC	Panel 16	protein microarrays	51.0	NA	82.0	0.730	<0.05
^101^	Mitchell (1990)	USA	(52/52)	25.0%	64.7 ± 9 (NA)	Serum	ADC(12) SCC(22) SCLC(7) Others(11)	Panel 17	ELISA	73.0	NA	NA	NA	<0.06
^103^	Zhong (2004)	USA	49/40	12 (30.0)	NA	Serum	ADC(12) SCC(19) Others(18)	Panel 18	ELISA	82.0	NA	83.0	0.837	0.0002
^44^	Chapman (2007)	Germany	82/50	9 (11.0)	63 (36–83)	Plasma	ADC(35) SCC(25) Others(22)	Panel 19	ELISA	76.0	NA	92.0	NA	<0.05
^98^	Qiu (2008)	USA	85/85	NA	NA	Serum	NSCLC	Panel 20	ELISA	51.0	NA	82.0	0.730	0.017

*AS* all-stage, *ES* early-stage (stage I and II included), *Controls* benign diseases and normal healthy donors, *AUC* area under the curve, *SEN* sensitivity, *SPE* specificity, *ELISA* enzyme-linked immunoassay, *WB* western blotting, *ADC* adenocarcinoma, *SCC* squamous carcinoma, *SCLC* small cell lung cancer, *NSCLC* non-small cell lung cancer, *NA* not available

Panel 1 (cyclin B1, Survivin, p53, HCCI)

Panel 2 (p37, p13, p10, p17, p12, p14, p15, p16, p22 and p1)

Panel 3 (cyclin B1, MDM2, c-Myc, p53, p16, 14-3-3ζ, NPM1)

Panel 4 (scFVB6, 3E, G1, J4, P6, J1)

Panel 5 (IMPDH, phosphoglycerate mutase, ubiquilin, Annexin I, Annexin II, HSP70-9B)

Panel 6 (CEA, CA-125, and CYFRA 21–1 antigens, anti-NY-ESO-1)

Panel 7 (p53, NY-ESO-1, CAGE, GBU4-5)

Panel 8 (p53, NY-ESO-1, CAGE, GBU4-5, Annexin 1, SOX2)

Panel 9 (M13 Phage 908, 3148, 1011, 3052, 1000)

Panel 10 (Phage peptide 72, 91, 96, 252, 286, 290)

Panel 11 (p53, GAGE7, PGP9.5, CAGE, MAGEA1, SOX2, GBU4-5)

Panel 12 (p62, BIRC, Livin-1, p53, PRDX, NYESO-1, ubiquilin)

Panel 13 (TTC14, BRAF, ACTL6B, MORC2, CTAG1B)

Panel 14 (p53, PGP9.5, SOX2, GAGE7, GBU4-5, CAGE, MAGEA1)

Panel 15 (p53, CAGE, NY-ESO-1, GBU4-5, Annexin I, SOX2, Hu-D)

Panel 16 (Annexin I, 14-3-3 Theta, LAMR1)

Panel 17 (MAb 5E8, IF10, and 5C7)

Panel 18 (BMI-1, p130, GAGE, HSP70, and HSP90)

Panel 19 (p53, c-myc, HER2 and CAGE)

Panel 20 (annexin I, 14-3-3 theta, and LAMR1)

## Study quality and characteristics

Study quality was evaluated by two reviewers (Yiyu Yin and Xiaoyan Li) independently. Any academic controversy was resolved by the following discussion among the researchers. All the studies in our research were of high quality with no risk of bias or the concern regarding their applicability, however, there were still unclear risks of bias and unclear applicability in patient selection and index tests in several studies. The statistics of the QUADAS-2 results of the 67 studies are shown in Table 3.

**Table 3:** Quality assessment of QUADAS-2

Reference	Study	Country	Domain 1: patient selection	Domain 2: index test(s)	Domain 3: reference standard	Domain 4: flow and timing	Score
^9^	Li (2017)	China	2	2	1	4	9
^10^	Dai (2016)	China	3	2	2	4	11
^44^	Chapman (2007)	Germany	3	2	2	3	10
^45^	Wang (2017)	USA	2	2	1	3	8
^79^	Dai (2017)	China	2	2	1	4	9
^63^	Mysikova (2016)	Czech Republic	3	1	2	3	9
^80^	Wang (2016)	China	3	1	1	4	9
^46^	Lui (2016)	USA	2	2	1	4	9
^64^	Yang (2015)	China	2	2	2	4	10
^81^	Qi (2015)	China	3	2	1	3	9
^82^	Lamy (2015)	France	3	1	1	3	8
^54^	Mattioni (2015)	Italy	2	2	1	4	9
^65^	Doseeva (2015)	USA	2	2	2	4	10
^83^	Wang (2014)	China	2	1	2	3	8
^84^	Ma (2013)	China	3	2	1	4	10
^85^	Pedchenko (2013)	USA	2	2	2	3	9
^86^	Dai (2013)	China	3	2	2	4	11
^47^	Ye (2013)	China	3	2	2	4	11
^69^	Zhang (2012)	China	3	2	1	4	10
^87^	Liu (2012)	China	3	1	2	4	10
^50^	Luo (2012)	China	2	2	1	4	9
^55^	Park (2011)	Korea	3	2	2	4	11
^41^	Khattar (2010)	USA	3	2	2	3	10
^42^	Wu (2010)	China	2	2	2	4	10
^88^	Yao (2010)	China	3	1	2	4	10
^48^	Cherneva (2010)	Bulgaria	2	1	2	4	10
^68^	Oji (2009)	Japan	2	1	1	4	8
^49^	Zhao (2018)	China	2	1	1	3	7
^89^	Pilyugin (2017)	Switzerland	3	1	2	3	9
^90^	Jung (2017)	Korea	2	2	1	4	9
^66^	Leidinger (2008)	Germany	2	2	2	4	10
^91^	Zhang (2017)	China	3	1	2	3	9
^52^	Wu (2018)	China	2	2	2	4	10
^43^	Wang (2015)	USA	3	2	2	3	10
^92^	Shen (2017)	China	2	2	2	4	10
^93^	Li (2016)	China	3	1	2	4	10
^53^	Costa (2014)	Netherlands	2	1	2	4	9
^94^	Dai (2017)	USA	3	1	1	4	9
^72^	Boyle (2010)	UK	2	2	2	4	10
^70^	Ren (2018)	China	3	2	2	4	11
^95^	Jia (2014)	China	3	2	1	4	10
^96^	Du (2018)	China	3	1	2	4	10
^97^	Chapman (2010)	UK	3	1	1	4	9
^98^	Qiu (2008)	USA	2	1	2	4	9
^71^	Farlow (2010)	USA	3	2	2	4	11
^71^	Surget (2013)	USA	3	2	2	3	10
^56^	Mack (2000)	Germany	2	2	2	3	9
^57^	Jerzy (1998)	Poland	2	2	2	3	9
^58^	Toshihiko (1998)	Japan	2	1	2	3	8
^99^	Oka (2001)	Japan	1	2	2	4	9
^59^	Jassem (2001)	Poland	2	2	2	3	9
^60^	Cioffi (2001)	Italy	2	2	2	4	10
^61^	Neri (2002)	Italy	2	2	2	4	10
^62^	Suleeporn (2003)	Thailand	1	1	2	3	7
^100^	Tsuji (1997)	Japan	1	2	2	3	8
^101^	Mitchell (1990)	USA	1	2	2	3	8
^102^	Dennis (2003)	USA	1	2	2	3	8
^103^	Zhong (2004)	USA	1	2	2	3	8
^84^	Zhong (2006)	USA	3	2	2	4	11
^98^	Ji (2008)	USA	1	2	2	4	9
^105^	Daniel (2008)	USA	1	2	2	3	8
^51^	Myrna (1997)	Germany	2	2	2	3	9

Each item was assessed as “yes” or “no” or “unclear”, and the score equaled to “1”, “0”, “0”, respectively. The full score of domain 1, domain 2, domain 3 and domain 4 was 3, 2, 2, 4, respectively. The total score of four domains greater than 7 was considered

A total of 67 studies are used in the case-control method in which every specimen was collected after LC diagnosis. Of the 67 studies, 52 analyzed single TAAbs (Table 1), 19 evaluated the performance of TAAbs panels (Table 2), 5 of which evaluated the diagnostic value of single TAABs and TAAbs panels at the same time^9,10,41–43^. Detailed information of each study on the number of cases and controls, mean or median age, specimen type, histological subtype, proportion of early-stage LC, detection method, and diagnostic indicators from each study are summarized in Tables 1 and 2.

Nearly all the included studies collected serum specimens except for 8 studies examined plasma^41,43–49^. Overall, the 67 studies evaluated 64 TAAbs and 20 TAAb panels in plasma or serum. The most commonly used detection method in studies of both single TAAb or with TAAbs panels, was enzyme linked immunoassay (ELISA), which was used in 52 out of 64 studies with single TAAbs and 19 out of 20 studies on TAAbs panels. The other detection methods used were Western blot (WB)^50,51^, Protein Chip^41^, serological spot assays^52^, sodium dodecyl sulfate polyacrylamide gel electrophoresis (SDS–PAGE), and liquid chromatography–electrospray mass spectrometry (LCMS)^53^. For the commercial panels of mixed TAAbs, the TAAbs were detected with ELISA.

## Diagnostic value of single TAAb at all stages of LC

We have listed the single TAAbs used to detect LC in Table 1. In the 52 studies covering 64 specific TAAbs, their sensitivities ranged from 3.1% to 92.9% (mean: 45.2%, median: 37.1%) and their specificities ranged from 60.6% to 100% (mean: 88.1%, median: 94.9%), the AUCs ranged from 0.416 to 0.990 (mean: 0.764, median: 0.785). However, the sensitivity of individual autoantibodies in 27 studies (51.9%) was lower than 50%. Twelve articles reported on the autoantibody against p53^9,10,51,54–62^, and found sensitivities ranging from 12.6% to 40.3% and specificities ranging from 94.9% to 100%. Three articles reported on the autoantibody against New York esophageal squamous cell carcinoma-1 (NY-ESO-1), and reported sensitivities from 26.3% to 47%, and specificities from 80.0% to 96.5%^63–65^. Two articles reported on the autoantibody against cyclin B1, with the sensitivities of 13.3% and 20%, and specificities of 96.6% and 97.6%^9,10^. The single TAAb with the most significant diagnostic value is the autoantibody against 27 Phage with the maximum sensitivity of 92.9% for SCC^66^.

## Diagnostic value of panels of TAAbs at all stages of LC

The diagnostic values of the 20 panels of TAAbs from 19 articles for all LC stages are listed in Table 2. Their sensitivities ranged from 30% to 94.8% (mean: 76.7%, median: 82%), their specificities ranged from 73% to 100% (mean: 86.8%, median: 89.0%), and their AUCs ranged from 0.630 to 0.982 (mean: 0.821, median: 0.820). In two articles, both of the sensitivity and specificity of TAAbs panels were >90.0%. These included panel 5 (IMPDH, phosphoglycerate mutase, ubiquitin, Annexin I, Annexin II, and HSP70-9B)^67^, and panel 10 (Phage 72, 91, 96, 252, 286, 290)^42^. The most significant AUC in panel 9 (M13 Phage 908, 3148, 1011, 3052, and 1000) was 0.982^42^.

## Diagnostic value of single TAAbs or panels of TAAbs for early-stage LC

The 11 specific TAAbs (including MUC1, NY-ESO-1, p53, APE1, CD25, CathepsinD, DKK1, WT1, 27Phage, TOPO48, and dickkopf-1 PepB) from 16 studies listed in Table 1. Their sensitivities ranged from 0% to 90.3% (mean: 41.2%, median: 39.3%), and their specificities ranged from 0% to 100% (mean: 91.8%, median: 95.3%). The TAAb with the most significant diagnostic value for detecting early stage LC is the autoantibody against Wilms tumor protein 1 (WT1) with a maximum sensitivity of 90.3% for NSCLC^68^.

The seven studies examining panels of TAAbs for detecting early stage LC were listed in Table 2. They show sensitivities ranging from 0% to 92.2% (mean: 58.3%, median: 62.0%), and specificities ranging from 79.5% to 92.2% (mean: 87.5%, median: 90.0%). Both the sensitivity and specificity in panel 10 (T7 Phage 72, 91, 96, 252, 286, 290) were above 90.0%^42^.

## Prospect of TAAbs as diagnostic biomarkers for LC

We performed a systematic review and identified 67 studies to evaluate the diagnostic performance of serum or plasma single TAAbs or TAAb panels for LC detection. From our results, we proposed that single or multiplex TAAbs may have diagnostic potential for both early stage or any stage of LC. Our results showed that although the great majority of individual TAAbs had low diagnositc sensitivities (Table 1), the TAAb panels supplied relatively high sensitivities, and some panels even had promising sensitivities and specificities (both >90%)^42,65^. In this present systematic review, our results comfirmed that the panel of 6 and 7 TAAbs had moderate diagnostic accuracy with mean AUCs of 0.850 and 0.806, respectively, at all LC stages, indicating that the diagnostic performance of the panel of six TAAbs at detecting LC was higher than that of the panel of seven TAAbs, However, the studies on the panel of six TAABs did not show any diagnostic values for the patients with early-stage LC except for only one study, which report a great sensitivity of 92.2%^42^.

Veronesi et al.^8^ reviewed the advances in LC-related markers, and found that the TAABs and miRNAs (MicroRNA) had great development potential for clinical detection and diagnosis of LC. However, they did not analyze the concrete diagnostic value of different single TAAbs or TAAb panels. Our systematic review found that different single and combinations of multiple TAAbs had different diagnostic performance for all stages of LC, and that more than half of the single TAAbs had low satisfactory diagnostic value with sensitivities lower than 50%. However,the panels of different TAAbs showed higher diagnostic performance with sensitivities ranging from 30.0% to 94.8% (mean: 76.7%, median: 82%), specificities ranging from 73.0% to 100.0% (mean: 86.8%, median: 89.0%), and AUCs ranging from 0.630 to 0.982 (mean: 0.821, median: 0.820). Doseeva et al.^65^ confirmed the value of using a mixed panel of tumor antigens and autoantibodies in the early detection of NSCLC in high-risk individuals. Their research showed that the use of NY-ESO-1 autoantibodies substantially increased the overall sensitivity of NSCLC detection. With the three tumor markers showing 77% sensitivity, 80% specificity, and a 0.850 AUC, while NY-ESO-1 alone only had 47% sensitivity, 80% specificity, and a 0.600 AUC. This was comfirmed by two studies by Zhang et al. and Park et al.^55,69^, which indicated that single TAAbs combined with other conventional markers (tumor antigens) were helpful at increasing the sensitivity and specificity for detecting LC. Therefore, while single TAAbs were barely capable of detecting LC at any stag with a high specificity and sensitivity, nevertheless their combinations with other markers could significantly improve their diagnostic value.

In our study, we summarized the studies on three panels^42,67,70^ containing six different TAAbs, two of which showed good sensitivities of 94.8% and 92.2% and specificities of 91.1% and 92.2%. Farlow et al.^71^ studied the panel of six TAAbs, which included inosine-5-monophosphate dehydrogenase (IMPDH), phosphoglycerate mutase, ubiquillin, Annexin I, Annexin II, and heat shock protein 70-9B (HSP70-9B), and found that its sensitivity for detecting LC was 94.8%. However, the study had a number of limitations, the first of which was that the sample size was too small, with only 10 cases in the experimental group, secondly, the adenocarcinoma was the only pathological subtype included. Therefore, the actual diagnostic value of this panel needs to be further verified. Wu et al.^42^ included 90 patients with NSCLC, and used an antigen panel of six TAAbs (phage peptide 72, 91, 96, 252, 286, 2906). Compared with the control group, the sensitivity was 92.2% and the specificity was 92.2%. In addition, they tested the serum of 21 early-stage NSCLC patients, and found that the sensitivity was aslo above 90%. They established a six phage peptides detector that could be used to diagnose early-stage NSCLC and discriminate between patients with NSCLC and patients with chronic obstructive pulmonary diseases (COPD). In order to make sure that the six phage peptide clones had high sensitivities and specificities for NSCLC, the researchers concentrated the NSCLC-specific phage peptide clones using biopannings. The 22 clones that had high reactivity with NSCLC but low reactivity with healthy control were selected for identification of the peptide targets, and the six highest immunoreactive phage clones were selected using individual serum samples of another 30 NSCLC patients. Hence, we indicated that panel of six TAAbs could probably be used to detect LC, especially at the early-stage in the near future. Another study by Boyle et al.^72^ did not report satisfactory results, with a sensitivity of only 37.0%. The antigens of the panel of six TAAbs they used were p53, NY-ESO-1, CAGE, GBU4-5, Annexin I, and SOX2, p53 is a tumor suppressor gene, which is the most frequently mutated gene in cancer (in addition to LC, it still can be found in breast cancer etc.^72^), indicating that it plays a crucial role in preventing cancer formation^73^. However, it can also be detected in some patients with chronic obstructive pulmonary disease (COPD)^7^. Therefore, TAAbs for p53 are nonspecific for LC detection. NY-ESO-1 is a cancer testis antigen, NY-ESO-1 appears to be expressed in 20–25% of NSCLC in most US studies, and SCC is more common in Japan while ADC is dominant in the United States and Europe^74^, stressing that different pathological subtypes may be involved and give clues to the basis of NY-ESO-1 expression in LC. CAGE is a cancer-associated gene, which expressed in a variety of cancers but not in normal tissues except the testis^75^, so it could be a target for antitumor immunotherapy. GBU4-5 is also a protein described as inducing autoantibodies in LC^76^. Annexin I, a phospholipid-binding protein has also been described as including autoantibodies, SOX2 was reported to induce autoantibody responses in SCLC^77,78^, indicating that autoantibodies to SOX2 could serve as good markers for SCLC, but are not appropriate for NSCLC. Most of the articles had high QUADAS-2 scores, showing that the overall methodological quality of most of the studies were good.

Low-dose CT screenings have the potential to detect early-stage LC and have demonstrated 20% lower LC mortality compared to chest X-ray screenings^78^. However, it is still difficult to detect LC in high-risk populations using only radiography. So identifying potential biomarkers, like TAAbs, that can be used to detect early-stage LC in a high-risk populations is urgently required, as they could have a distinctly beneficial and clinically significant impact on patient survival^12^. In our systematic review, several studies were included that reported on single or combinations of multiple TAAbs for detection of early-stage LC. For single TAAbs, the sensitivity for early-stage LC ranged from 0% to 90.3% (mean: 41.2%, median: 39.3%), and the specificities ranged from 0% to 100% (mean: 91.8%, median: 95.3%). One study reported that the autoantibody against Wilms tumor protein 1 (WT1) had the maximum sensitivity of 90.3% for NSCLC^68^. The sensitivities of TAAb panels at detecting early-stage LC patients ranged from 0% to 92.2% (mean: 58.3%,median: 62.0%), and their specificities ranged from 79.5% to 92.2% (mean: 87.5%, median: 90.0%). Although the sensitivities in most of the included studies were below 50.0%, in a study conducted by Wu et al.^42^, six cancer-associated proteins (Phage peptide 72, 91, 96, 252, 286, and 290) were used as markers of LC with a maximum sensitivity of 92.2% and specificity of 92.2% in 21 patients with stage I–II NSCLC. However, the sensitivity of a seven TAAbs panel (cyclin B1, MDM2, c-Myc, p53, p16, 14-3-3ζ, and NPM1), was 73.3% and its specificity was 79.5%, the panel of CEA, CA-125, and CYFRA21-1 antigens, and NY-ESO-1 antibody, had a sensitivity of 71.2%, in addition, the seven TAAb panels (p53, GAGE7, PGP9.5, CAGE, MAGEA1, SOX2, and GBU4-5), (p53, PGP9.5, SOX2, GAGE7, GBU4-5, CAGE, and MAGEA1), (p53, CAGE, NY-ESO-1, GBU4-5, Annexin I, SOX2, and Hu-D) had sensitivities of 62.0%, 56.4%, and 53.0%, respectively. In conclusion, the diagnostic value of the panel of six TAAbs seems to be higher than the panels of seven TAAbs.

Our study has some deficiencies. First, we just searched Pubmed and ISI Web of Science for articles published from 1 January 1990 to 31 December 2018, which may not cover the all relevant studies. Second, we defined stage I LC as early-stage, and a few studies included did not report the exact number of the patients with stage I LC, but stage I–II instead, which may cause some publication bias. Third, the studies included used different methods, which may influence our results. Although some studies did find great diagnostic value for LC, the diagnostic TAABs still cannot be used alone in a clinical setting, as they must be integrated with low-dose CT scan imaging in the screening procedure.

## Conclusion

Our study indicated that single TAAbs or TAAb panels may be useful biomarkers for detecting LC patients at all stages or specifically early-stage LC in high-risk populations or healthy people, but the TAAb panels showed a higher diagnostic performance than single TAAbs. The diagnostic value of the panel of six TAAbs is higher than the panels of seven TAAbs, and may be used as potential biomarkers for the early detection of LC and in combination with low-dose CT can probably be used in clinical settings^79–105^.
